# Extraction and Evaluation of Corpus Callosum from 2D Brain MRI Slice: A Study with Cuckoo Search Algorithm

**DOI:** 10.1155/2021/5524637

**Published:** 2021-08-01

**Authors:** K. Suresh Manic, Roshima Biju, Warish Patel, Muhammad Attique Khan, N. Sri Madhava Raja, S. Uma

**Affiliations:** ^1^Department of Electrical and Communication Eng., National University of Science and Tech, Muscat, Oman; ^2^Research Scholar, Department of Computer Science Eng., Parul University, Vadodara, Gujarat 391760, India; ^3^Department of Computer Science Eng., Parul University, Vadodara, Gujarat 391760, India; ^4^Department of Computer Science, HITEC University, Museum Road, Taxila, Pakistan; ^5^Department of Electronics and Instrumentation Eng., St. Joseph's College of Engineering, OMR, Chennai 119, India; ^6^Research Scholar, Anna University, Chennai, India

## Abstract

The work proposes a computer-based diagnosis method (CBDM) to delineate and assess the corpus callosum (CC) segment from the 2-dimensional (2D) brain magnetic resonance images (MRI). The proposed CBDM consists of two parts: (1) preprocessing and (2) postprocessing sections. The preprocessing tools have a multithreshold technique with the chaotic cuckoo search (CCS) algorithm and a preferred threshold procedure. The postprocessing employs a delineation process for extracting the CC section. The proposed CBDM finally extracts the vital CC parameters, such as total brain area (TBA) and CC area (CCA) to classify the considered 2D MRI slices into the control and autism spectrum disorder (ASD) groups. This attempt considers the benchmark brain MRI database which includes ABIDE and MIDAS for the experimental investigation. The results obtained with ABIDE dataset are further confirmed against the fuzzy *C*-means driven level set (FCM + LS) and multiphase level set (MLS) technique and the proposed CBDM with Shannon entropy along with active contour (SE + AC) presented improved result in comparison to the existing methodologies. Further, the performance of CBDM is confirmed on MIDAS and clinical dataset. The experimental outcomes approve that the proposed CBDM extracts the CC section from the 2D MR brain images that have higher accuracy compared to alternative techniques.

## 1. Introduction

Corpus callosum (CC) is one among the vital brain parts responsible for neural communication among the two brain sections. CC is the prime commissural territory in the human brain, and it is composed of nearly 200-300 axons [1]. The work by Hinkley et al. (2012) on agenesis of corpus callosum (ACC) confirms that CC plays a significant role in problem cracking schemes and swiftness in vocal processing [2]. The study of Paul et al. (2014) also presents the relation of ACC and autism [3]. Their work also confirms that the CC disorder will lead to autism. In early significant researches, many works are reported to observe autism disorder based on CC [4–7]. Some of similar research works also report the study of sexual dimorphism in CC [8–14].

Due to its clinical significance, a substantial amount of CC assessment procedures has been proposed and discussed by researchers [15, 16]. Normally, the CC region is best visible in the sagittal view of two-dimensional (2D) brain MRI. The visibility of the CC is also approximately similar to other normal brain tissues and hence, the segmentation of CC from the MRI requires some complex procedures in comparison with the separation of other brain regions. Considering literature, procedures such as manual segmentation [6], level set scheme [10], active contour method [15], and fuzzy *C*-means [16] are applied to extract CC with possible accuracy. Most of these approaches consider a two-step procedure to separate the CC from the sagittal view MRIs.

In recent times, the two-step process which integrates multithresholding and segmentation is widely adopted by the investigators to obtain the region of interest (ROI) of the brain MRI documented with various modalities such as flair, T1, T1C, T2, and diffused weighting (DW) [17–22]. These approaches implement the heuristic algorithm-oriented threshold process to develop the prominence of the ROI and a preferred segmentation plan to mine the ROI. Further, the ROI is assessed in comparison with the corresponding ground truth (GT) pictures presented by a domain professional. The image similarity parameters (ISP) obtained during the ROI and GT evaluation confirm the superiority of the brain MRI assessment technique [23–25].

The earlier works confirms that the heuristic approach-based brain MRI work offers improved result. This is obtained in comparison with the existing conventional processes [25]. Hence, in this work, most successful heuristic procedure called the cuckoo search (CS) algorithm is considered during the brain MRI preprocessing. The performance of the traditional CS (TCS) is enhanced based on the chaotic operator known as Ikeda map (IM), which aided to accomplish better threshold result. The details of the IM and its application are discussed in [20, 21). Experimental investigation of their work is then compared with the Lévy-Flight and Brownian-Walk operators, which confirmed that the chaotic cuckoo search (CCS) offers better threshold compared to the traditional CS.

During the preprocessing procedure, CCS identifies the optimal value of thresholds for brain MRI. In preprocessing, a comparative examination amongst the famous threshold approaches, such as Kapur, Tsallis, Otsu, and Shannon is performed. This helps in finding the best suited threshold scheme for CC examination using the 2D brain MRI. The role of the postprocessing plan is to demarcate the CC subjected to preprocessing. After mining the CC, an assessment in comparison with the ground truth is performed to obtain the vital ISPs.

In literature, few methods are discussed to obtain the CC present in the considered 2D brain images. Further, most of the methods are interested in computing the total brain area (TBA) and corpus callosum area (CCA) to categorize the 2D brain MRI dataset into control and autism spectrum disorder (ASD) groups. In analyzing an image which belongs to medical, it is always essential to measure the outcome of the proposed tool with a chosen image dataset. If the tool works well on the dataset, further, the developed image examination instrument can be considered to estimate the medical grade images.

The earlier works on CC examination computes only the TBA and CCA and directly implements a categorization process. To evaluate the efficacy of the developed tool, it is essential to compute the ISPs and the essential statistical measures. Further, the soft computing-based CC examination is also needed to improve the extraction accuracy. Because of these reasons, in the projected work, CCS with CBDM is proposed for examining the CC section.

This research work also presents a detailed study on (i) different threshold procedures, such as Otsu, Kapur, Tsallis, and Shannon and (ii) various segmentation approaches, such as level set (LS), Chan-Vese (CV), region growing (RG), and active contour (AC) in order to identify the appropriate pre- and postprocessing practice to mine CC.

The experimental investigation is implemented in Matlab software (Version7, Release14, Lic. No. 285705 with perpetual term) using the public autistic databases, like ABIDE (images of 60 volunteers) [19, 20] and MIDAS (images of 4 × 2 volunteers) [21]. The clinical implication of projected tool is confirmed with the real-time clinical MRI obtained from Proscans laboratory (images of 10 × 2 volunteers) [22].

## 2. Related Works

**Figure 1:**
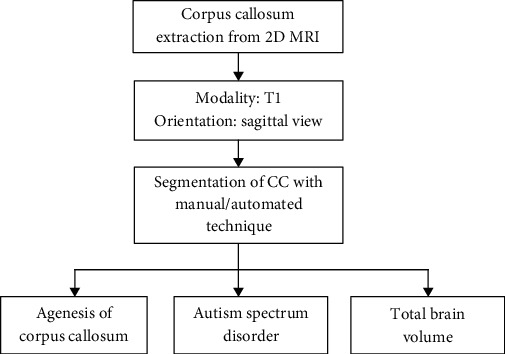
Various approaches to examine the CC to detect different brain abnormalities.

The MR imaging technique is extensively utilized to record the performance and malformations of internal organs of living beings. The improvement in the MRI method additionally supports the upgrading and appraisal of features recorded in 3-dimensional digital pictures. Prevailing evaluation methods which deal with 3D images are intricate. They require extraordinary swiftness in computing machines since data volume is enormous. To reduce the difficulty in assessing the MRI, reorganized 3D image is further transformed into a significant amount of 2D slices. Finally, the two-dimensional slices are assessed using a suitable image investigation system. In the proposed work, 2D brain MRI slices are considered for the study, and the stages involved in the CC segmentation and the corresponding brain abnormalities to be detected are presented in Figure 1.

Examination of CC from 2D slices of MRI with sagittal view is commonly considered by the researchers. Paul et al. (2014) proposed a practical examination to compare ACC and autism using 2D MRI of T1 and DW modalities [3]. Their work confirms that examination of CC is essential to asses ACC and autism. Wolff et al. (2015) proposed a clinical investigation to confirm that the CC region is reduced for elders and adults having autism spectrum disorder (ASD) [4]. Frazier and Hardan (2009) applied a region-based examination on CC section of patients with autism [5]. A manual segmentation scheme is considered to extract and evaluate the CC's size values and confirmed that the ASD can be predicted based on the size of the CC section. Tepest et al. (2010) inspected the size of CC and its segments associated with total brain volume (TBV) to identify the autism with respect to gender and revealed that the TBV values in males are higher than in females [6]. Lefebvre et al. (2015) proposed a work on neuroanatomical variety of CC and TBV in autism and verified the work by considering the brain MRIs of 694 volunteers [7]. The studies on the sexual dimorphism in CC and variation in size of TBV and CC also widely examined using the 2D MRI slices [8].

Previous studies authenticate the requirement of CC examination during the human brain analysis; hence, more care is essential during the segmentation of the CC region. Normally, the CC is a thin section in the brain MRI and will have the pixel intensity similar to other brain sections. Hence, it is essential to consider an efficient image processing system to excerpt and estimate the CC from the 2D brain MRI of a chosen modality.

Fredo et al. (2014) applied a two-step process with fuzzy-C-means (FCM) clustering and multiphase level set (LS) approach to delineate the CC, cerebellum, and brain stem from the 2D MRI recorded with T1 modality and obtained a mean area of 0.87 for control (normal) cases and 0.67 for ASD cases [9]. Further, Fredo et al. (2015) implemented a similar work on the ABIDE database with 20 samples of control cases (male = 14 and female = 6) and 20 samples of ASD cases (male = 11 and female = 9) and attained mean area of 0.90 for control cases and 0.75 for ASD [10, 11]. Fredo et al. (2015) employed the reaction diffusion regularized level set (RDRLS) method to delineate CC [10]. Vachet et al. (2012) implemented the deformable active Fourier contour model [15], and İçer (2013) discussed a two-step approach based on the Gaussian mixture model and FCM to extract the CC [16]. Li et al. (2013) executed an automated two-step segmentation scheme by combining the mean shift clustering technique-based image improvement and geometric active contour (GAC) dependant segmentation of CC [26]. The work of Elsayed et al. (2010) implements a spectral segmentation with the multiscale graph decomposition process to extract CC [27]. Recent review of Cover et al. (2018) presents an elaborate evaluation of various CC evaluation schemes, MRI modalities, and performance measures existing in the literature [28]. Their work also reports that T1-weighted MRI is the widely adopted modality (44%) to examine CC. This work also discusses the merits and demerits of the existing schemes and also recommends the need for a novel evaluation tool.

The proposed procedure has a two-stage process to extract the CC section present in 2D brain MRI of T1 modality that is also implemented. For experimental investigation, the database such as ABIDE and MIDAS is utilized. Further, in this proposed method, it is implemented and validated for the clinical MR image obtained from Proscans laboratory.

## 3. Computer-Based Diagnosis Method

A brief summary concerning the methods adopted in this paper to provide a computer-based diagnosis method (CBDM) for the extraction of CC from 2D brain MRI is discussed in this segment. The outline of the database utilized, rudimentary tasks in examining images of brain, preprocessing, delineation, and validation are presented elaborately.

**Figure 2:**
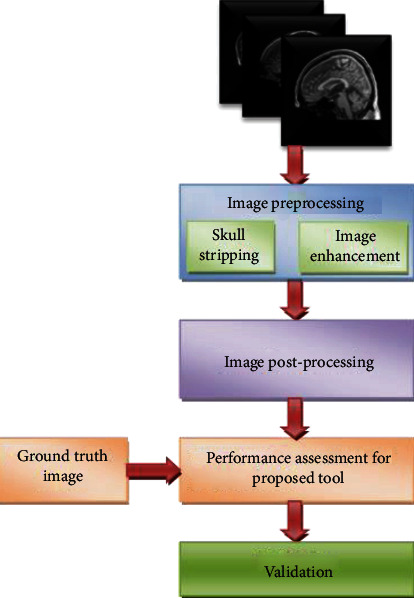
Overview of proposed tool.

Figure 2 presents various phases used in this examination tool. Firstly, a 2D image of sagittal view MRI of chosen slice is considered with/without skull section. Early improvement of raw MRI slice is carried out using an appropriate image preprocessing method, and a preferred postprocessing scheme is to be adopted to mine the CC section. Further, the performance of the tool can be validated with a relative analysis with ground truth (GT) image presented by a specialist. Extracted CC is then validated by a doctor by providing the decision of next step in treating the patient to normalize or provide remedy for the brain prognosis state. In all cases, the developed tool by any suitable approach can only offer a suggestion/preopinion regarding the brain abnormality, and the doctor has to provide consultation and thereby a conclusion in treating the patient further based on the condition.

### 3.1. MRI Database

The sagittal view of MRI adopted in this paper is collected from the public database such as ABIDE and MIDAS. Further, real clinical image obtained from Proscans is also used in this research work. These entire databases consist of 3D brain structures recorded with T1 modality. ITK-SNAP version 3.6.0 tool aids to obtain the 2D slices from the complete dataset. [29, 30], and an image normalization is implemented to obtain 2D slices of size 256 × 256 pixels. Similar practice is employed for the GT image of ABIDE. The ABIDE is the commonly used database in autism studies, which provides the vital details, like subject case (controlled/autistic), gender, age, area of CC, and TBV [9–11].

In this work, 60 volunteer's (age group of 13-16 years) images are considered for the examination. The MIDAS database consists of two control (normal) and two autistic volunteer's images that are recorded in the age of 2 years and follow-up in 4 years, respectively. Finally, the clinical images of a volunteer collected from Proscans are also examined using the proposed approach.

### 3.2. Image Preprocessing

This scheme is generally considered to improve the picture under assessment using a suitable image processing technique. This procedure will increase the ROI by uniting the similar pixel values with a set of threshold values selected. Recent related works confirm that preprocessing practice is an essential stage in two-step image processing tool.

#### 3.2.1. Skull Stripping

Usually, the reconstructed brain MRI is associated with the outer head bone called the skull. For modalities of T2 and flair type, concentration of pixels belonging to the skull is roughly greater than soft brain tissues. Also, in T1 MRI modality, the skull intensity is similar to the intensity level of the brain tissue. Automated brain region segmentation always requires a suitable skull stripping procedure to discrete the soft region of the brain from that of the skull information [31]. Various skull removing methods discussed by the researchers can be found in [32]. If semiautomated brain section segmentation is implemented, the additional procedure of skull removal will not be required further. The methods such as LS, CV, RG, and AC fall in the semiautomated group do not take into consideration of the skull section. In this paper, the work is instigated on the 2D MRI slice, with and without skull section.

#### 3.2.2. Cuckoo Search Algorithm

In consideration with various existing metaheuristic methods, cuckoo search (CS) presented by Yang and Deb has appeared to be one of the effective soft computing techniques [33–37]. Recently, CS is widely accepted by maximum number of researchers in solving numerous optimization tasks [38, 39]. The main advantage of CS compared with the firefly and bat algorithm is the structure of the CC that is simple and supports higher probability of getting the optimized solution. Various chaotic search procedures assisted CS can be found in [40, 41].

The mathematical expression of CS is as follows:

The CS executed with the following conventions:

- Each bird leaves behind an egg in randomly nominated nest of other host birds
- Nest of strong surviving egg is inherited to the succeeding level. The hatching rate of this egg is faster than that of the host
- The chance of categorizing the egg by host bird in CS is *p*_*a*_ ∈ [0, 1] for a selected optimization task

In most of the heuristic algorithms, accomplishment in discovering a resolution for a job generally depends on its direction finding method. Typically, it is guided by Lévy Flight (LF) and chaotic strategies [42, 43].

In this paper, the Ikeda map (IM) is chosen to drive the CS, and the details on IM are available in [44, 45]. In CS optimization investigation, nascent location (*X*^(*t* + 1)^) naturally count on previous position (*X*_*i*_^(*t*)^). In this section, the subsequent equations are accounted to search an updated location of cuckoo:

(1) $$\begin{array}{c} X_{i}^{\left(t+1\right)}=X_{i}^{\left(t\right)}+\alpha ⊕\text{IM}, \end{array}$$

where *X*_*i*_^(*t* + 1)^ is the updated position and signifies early position, **⊕** denotes the entrant multiplier, and IM shows the chaotic Ikeda map approach. Normally, the parameter “*α*” is assigned with a positive integer (i.e., *α* > 0) and in this research, “*α*” is allocated as 1. Additional particulars regarding CS are cited in the works of Yang and Deb [37].

IM is one of the chaotic search operator, and its explanation and application on various heuristic algorithms can be found in [17, 18].

(2) $$\begin{array}{c} \text{IM}=R..\exp\left[Z\Phi -Z\left(\frac{\delta }{1+{\left|{X_{i}}^{\left(t\right)}\right|}^{2}}\right)\right], \end{array}$$

where *Z* is the iteration number, *ϕ* is allotted as 0.1, *δ* is chosen as 7, and the disordered attraction constraint (*R*) value is given as 0.75.

Equation (2) presents the IM implemented in the recent attempt of Satapathy et al. (2018) to increase the investigation competence of the bat algorithm (BA) [46]. This work established that the IM-assisted BA offered better result in comparison with particle swarm optimization (PSO), firefly algorithm (FA), and traditional BA. Further, the work of Abhinaya and Raja (2015) [17] and Lakshmi et al. (2016) [18] confirms the advantage of IM-based cuckoo search for the medical image processing. Hence, this work implements a chaotic IM search technique to improve the performance of the traditional cuckoo search (TCS) method. The efficacy of the proposed chaotic cuckoo search (CCS) is further confirmed with other techniques, such as particle swarm optimization (PSO) [47], bacterial foraging optimization (BFO) [48], bat algorithm (BA) [41], and TCS [41].

The subsequent initial constraints are assigned for every heuristic algorithms adopted in this paper: representative's dimension is designated as 30, exploration measure is set as 3 (a three-level), the complete iteration limit is maintained as 1500, and end criteria are given as maximized value of image measure (between class variance for Otsu and maximized entropy for Kapur, Shannon, and Tsallis thresholding schemes).

#### 3.2.3. Image Thresholding

Thresholding is an extensively followed image enhancement process employed to process traditional and medical images [23–25]. During the threshold process, a picture frame is separated into several sections by grouping related pixels, to find and evaluate the significant information existing in the picture. Previous research works confirm the availability of a variety of threshold schemes, such as Otsu, Kapur, Shannon, and Tsallis to preprocess the gray scale and RGB pictures. This section also implements a comparative study among the above said threshold procedures.

*(1) Otsu's Scheme*. This scheme is one of the well-known procedures widely adopted to progress the trial picture based on the chosen threshold value. In the related works of this research, multithresholding based on Otsu's approach is widely applied by the researchers for a class of image cases based on maximizing the interclass variance.

Otsu is a nonparametric threshold scheme developed in 1979 [49], and its mathematical relation is depicted as follows;

Let *L* = 256, and the chosen threshold number is three (i.e., Th = 3), which divides the input image into three distinct groups, like *Q*0, *Q*1, and *Q*2.

Assume that the image consists the thresholds like (*t*_1_, *t*_2_ ⋯ *t*_Th_), which split the input picture into three groups: *Q*0; gray level values are accounted from 0 to *t* − 1, *Q*1 which has gray levels of range *t*_1_ to *t*2 − 1, and *Q*2 contains gray levels from *t*3 to *L* − 1.

The objective function for the above case will be

(3) $$\begin{array}{c} \mathrm{Maximize}\;F\left(T\right)=\varphi_{0}+\varphi_{1}+\varphi_{2}, \end{array}$$

where *φ*_0_ = *ω*_0_( *μ*_0_ − *μ*_*T*_)^2^, *φ*_1_ = *ω*_1_( *μ*_1_ − *μ*_*T*_)^2^, *φ*_2_ = *ω*_2_( *μ*_2_ − *μ*_*T*_)^2^.

In Eq. (3), the symbols *ω* and *μ* represent the class probabilities and class means, respectively.

*(2) Kapur's Technique*. Kapur's entropy (KE) was originally proposed in 1985 to appraise gray scaled images in accordance with its entropy based on histogram [50]. KE aids to explore the optimal threshold of a picture on the basis of its entropy alone. Since the outcome proves to provide satisfactory results, many researches using KE are deliberated in the literature [18].

Precise model of the KE is well defined as follows:

Let *T* = [*t*_1_, *t*_2_, ⋯, *t*_*L*−1_] represent individual threshold values of the image. Further, the complete entropy of KE is represented as follows:

(4) $$\begin{array}{c} \text{Costfunction}=J_{\text{Kapur}}=F\left(T\right)=\overset{L}{\underset{J=1}{\sum }}O_{j}^{R}\text{for}R\left\{1,2,3\right\}. \end{array}$$

Equation (4) designates to get the most out of value of entropy for the selected threshold.

In trilevel thresholding assignment, the objective function value is denoted as

(5) O1R=∑j=11tPojRθ0RlnPojRθ0R,O2R=∑lj=t+12tPojRθ1RlnPojRθ1R,O3R=∑2j=t+1LPojRθ2RlnPojRθ2R,

where *Po*_*j*_^*R*^ shows the likelihood distribution and *θ*_0_^*R*^, *θ*_1_^*R*^, .*θ*_2_^*R*^ depicts the probability occurrence in *L*-levels.

*(3) Shannon's Technique*. Shannon's entropy (SE) procedure was established by Kannappan in 1972 [51]. Rajinikanth et al. (2017) states that the SE approach-based brain MRI examination offers better result in comparison with Kapur's and Tsallis technique [18].

In recent works, SE dependant thresholding is employed to perform preprocess medical pictures. To elucidate the SE, a picture with dimension *A* × *B* is to be under consideration. The pixel arrangement of the gray picture (*h*, *v*) is expressed as *G*(*h*, *v*), for *h* ∈ { 1, 2, ⋯, *A*} and *v* ∈ { 1, 2, ⋯, *B*}. Let *L* be the various levels of gray for the considered test image, and the set of all gray values {0, 1, 2, ⋯, *L* − 1} can be symbolized as *Z*, in such a way that

(6) $$\begin{array}{c} G\left(h,v\right)\in Z\forall \left(h,v\right)\in \text{picture}. \end{array}$$

Then, the normalized histogram will be *X* = {*t*_1_, *t*_2_, ⋯, *t*_L−1_}.

For thresholding with level set to 3, Eq. (5) becomes

(7) $$\begin{array}{c} X\left(T\right)=x_{0}\left(t_{1}\right)+x_{1}\left(t_{2}\right)+x_{2}\left(t_{3}\right),\\ F\left(T\right)=\underset{T}{\max}\left\{X\left(T\right)\right\}. \end{array}$$

Threshold value which is represented by *T* = {*t*_1_, *t*_2_, ⋯, *t*_*L*_}, *X* = {*x*_0_, *x*_1_, ⋯, *x*_*L*−1_} denotes the normalized histogram, and *F*(*T*) indicates the optimal threshold. Further information about SE can be found in [52].

*(4) Tsallis Technique*. Tsallis entropy (TE) is a nonextensive entropy idea derived from the SE by Tsallis [53, 54] and represented as

(8) $$\begin{array}{c} S_{q}=\frac{1-\sum_{i=1}^{T}{\left(p_{i}\right)}^{q}}{q-1}. \end{array}$$

In the equation, *T* is the scheme prospective, *q* is the entropic indicator, and *pi* represents the probability of each state *i*. Usually, the entropy value obtained with Tsallis procedure, *Sq*, will meet Shannon's entropy when *q*⟶*l*.

The entropy information is denoted using a quasiadditive instruction as

(9) $$\begin{array}{c} S_{q}\left(A+B\right)=S_{q}\left(A\right)+S_{q}\left(B\right)+\left(1-q\right).S_{q}\left(A\right).S_{q}\left(B\right). \end{array}$$

TE can be utilized to discover the finest threshold values in the image. A test picture with *L* gray levels which have the values {0, 1, ⋯, *L* − 1} with possibility spreading *p*_*i*_ = *p*_0_, *p*_1_, ⋯, *p*_*L*−1_ is considered. Thus, the Tsallis trilevel-based threshold process is achieved with the objective function:

(10) $$\begin{array}{c} F\left(T\right)=\left[t1,t2,t3\right]=\text{argmax},\\ F\left(T\right)=\left[t_{1},t_{2},t_{3}\right]=\text{argmax},\\ \left[S_{q}^{A}\left(T\right)+S_{q}^{B}\left(T\right)+S_{q}^{C}\left(T\right)+\left(1-q\right).S_{q}^{A}\left(T\right).S_{q}^{B}\left(T\right).S_{q}^{C}\left(T\right)\right], \end{array}$$

where

(11) SqAT=1−∑i=01t−1Pi/PAq−1q,PA=∑i=01t−1Pi,SqBT=1−∑i=t1t2−1Pi/PBq−1q,PB=∑i=t1t2−1Pi,SqCT=1−∑i=t2L−1Pi/PCq−1q,PC=∑i=t2L−1Pi.

When the multilevel process is executed base on threshold, an optimal threshold value *T* is to be obtained such that the objective function *F*(*T*) is being maximized. In this existing work, the principal part of the CCS algorithm is to discover the maximized optimal threshold “*F*(*T*)” in Otsu, KE, SE, and TE cases for a chosen threshold of three.

### 3.3. Image Postprocessing

This phase purpose is to mine the ROI (CC) from preprocessed brain MRI. The details of various automated and semiautomated separation measures prevailing in the image processing literature are presented in detail. Based on the implementation, the segmentation processes are categorized as (i) automated and (ii) semiautomated schemes. In the automated scheme, the segmentation procedure requires a minimal or nil operators' assistance. In the semiautomated method, the initiation of the segmentation task is to be done by the operator based on a trial and error approach or a by adopting a directed practice.

#### 3.3.1. Automated Segmentation

The segmentation methods, such as watershed [55], principal component analysis [56], and clustering approaches (*k*-means, fuzzy *k*-means, etc.) [57], are some of the techniques that falls in the category of the automated segmentation approach. In these procedures, the interaction of human operator during the initiation is comparatively less.

#### 3.3.2. Semiautomated Segmentation

Semiautomated segmentation (SAS) approaches are widely considered in medical image analysis, when a complex segmentation task is to be completed. In these methods, the operator's assistance is essential throughout the segmentation execution. The operator is responsible to begin the operation, assigning the run time/number of iteration required and assigning the terminating criterion. SAS is widely applied by the investigators to extract the ROI from a class of complex medical images [25]. Generally, SAS works based on the identification of the similar pixel values from its initial point. It will explore all the possible alike pixel values present in the preprocessed picture, until the maximum iteration value is reached. The approaches, such as level set (LS) [58], Chan-Vese (CV) [59, 60], region growing (RG) [61], and active contour (AC) [62], fall in this category. In the projected work, the AC segmentation is executed to obtain the CC, and its performance is then validated against alternative approaches, like LS, CV, and RG.

AC has an adaptable snake-like search mechanism, which modifies its direction such that it addresses all the possible comparable pixel clusters available in the image based on energy minimization theory as discuss in [63]. Because of its merit, AC is commonly adopted to inspect medical images.

AC performs operations, like (i) border recognition, (ii) preliminary curve generation with respect to the identified border, (iii) changing the snake's orientation to follow the pixel group till the energy becomes minimal, and (iv) final curve generation and extraction of the region inside the final contour.

Energy function of AC's snake is

(12) $$\begin{array}{c} \frac{\min}{C}\left\{E_{\text{GAC}}\left(C\right)=\int_{0}^{L\left(C\right)}g\left(\left|\nabla I_{0}C\left(s\right)\right|\right)ds\right\}, \end{array}$$

where *ds* is the Euclidean distance constituent and *L*(*C*) is the length of the curvature *C*. It satisfies the constraints *L*(*C*) = ∫_0_^*L*(*C*)^*ds*. The limitation *g* indicates edge, which will wane based on the objective periphery defined as

(13) $$\begin{array}{c} g\left(\left|\nabla I_{0}\right|\right)=\frac{1}{1+\beta {\left|\nabla I_{0}\right|}^{2}}, \end{array}$$

where *I*_0_ signifies test image under study and *β* depicts a random constant. The energy value quickly declines because of the values reflected by the edges as in gradient succession quantification.

This method is scientifically characterized as

(14) $$\begin{array}{c} \partial_{t}C=\left(kg-\langle \nabla_{g},M\rangle \right)M, \end{array}$$

where*∂*_*t*_*C* = *∂C*/*∂t* indicates the changes in the snake model. *t* represents the repetition period. *k* and *M* are the curve and normal for the considered snake “*C*.” In this process, the silhouette of the snake is constantly adjusted till nominal value of the energy; *E*_GAC_ is accomplished.

### 3.4. Evaluation of ROI with GT

The goal of this section focuses to examine the performance of the suggested method by employing a qualified examination amongst ROI and GT. This work deliberates standard brain MRI dataset identified as ABIDE, in which test images are associated with GT. In this study, image resemblance values, such as Jaccard, dice, false-positive rate (FPR), and false-negative rate (FNR), are computed [23–25].

The mathematical terminologies are presented in Eqs. (15)–(18):

(15) $$\begin{array}{c} \text{Jaccard}\left(I_{G},I_{C}\right)=I_{G}\cap I_{C}I_{G}\cup I_{C}, \end{array}$$

(16) $$\begin{array}{c} \text{Dice}\left(I_{G},I_{C}\right)=2\left(I_{G}\cap I_{C}\;\right)\left|I_{G}\right|\cup \left|I_{C}\right|, \end{array}$$

(17) $$\begin{array}{c} \text{FPR}\left(I_{G},I_{C}\right)=\left(I_{G}I_{C}\right)\left(I_{G}\cup I_{C}\right), \end{array}$$

(18) $$\begin{array}{c} \text{FNR}\left(I_{G},I_{C}\right)=\left(I_{C}I_{G}\right)\;\left(I_{G}\cup I_{C}\right), \end{array}$$

where *I*_*G*_ signifies the GT and *I*_*C*_ represents the mined section.

Furthermore, the image statistical outcomes, which include sensitivity, specificity, accuracy, and precision, are also calculated [64, 65].

Expressions for these bounds are specified in Eqs. (19)–(22):

(19) $$\begin{array}{c} \text{Sensitvity}=T_{P}\left(T_{P}+F_{N}\right), \end{array}$$

(20) $$\begin{array}{c} \text{Specificity}=T_{N}\left(T_{N}+F_{P}\right), \end{array}$$

(21) $$\begin{array}{c} \text{Accuracy}=\left(T_{P}+T_{N}\right)/\left(T_{P}+T_{N}+F_{P}+F_{N}\right), \end{array}$$

(22) $$\begin{array}{c} \text{Precision}=T_{P}\left(T_{P}+F_{P}\right), \end{array}$$

where *T*_*N*_, *T*_*P*_, *F*_*N*_, and *F*_*P*_ signify related measures.

## 4. Result and Discussions

The outcomes accomplished with the planned tool are elaborated. Various early works endorse the accessibility of considerable processing procedures for CC examination of the considered images. The projected work tools have a two-stage procedure to observe the well-known 2D sagittal brain MRI and the MR images obtained from the clinic. This work reflects the support of the contemporary heuristic technique known as CCS along with the well-known threshold approach. A comprehensive valuation among the prevailing segmentation processes, such as LS, CV, RG, and AC, is also presented. The developed CDT is executed with a AMD C70 Dual Core 1 GHz CPU with 4 GB of RAM PC which is equipped with Matlab software.

Firstly, the ABIDE dataset of 60 volunteers (30 control and 30 ASD class) is considered for the examination. This database contains the 2D sagittal MRI recorded with T1 modality with a pixel measurement of 256 × 256. This dataset is associated with relevant GT offered by a professional.

**Figure 3:**
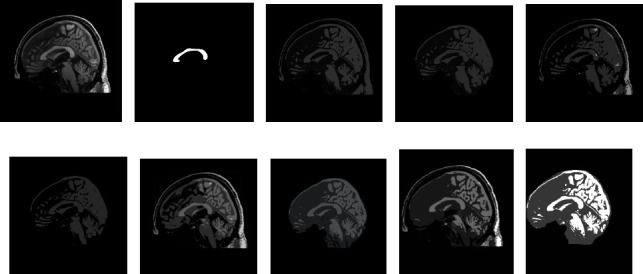
Outcome of the preprocessing approach for a chosen test image (image 1). (a, b) depicts test image and the GT, respectively, (c, d) shows Otsu's thresholding outcome for image with and without skull section, (e, f) presents the outcome of Kapur's entropy, (g, h) depicts the thresholding result of Shannon's, and (i, j) illustrates the result by Tsallis.

Figure 3 depicts a chosen 2D MRI and the GT of ABIDE. The preprocessing procedure is then implemented on this image by considering its original version and the skull stripped version. This figure also depicts the threshold results of various procedures reflected in this work. Figures 3(c) and 3(d) represent the enhanced image with Otsu's approach, Figures 3(e) and 3(f) depict the outcome of KE-based trilevel thresholding, and Figures 3(g) and 3(h) show that the result of SE and Figures 3(i) and 3(j) shows the results by TE. After enhancing the test picture based on a chosen threshold approach, a segmentation task is used to extract the CC section in order to find the parameters, such as TBA and CCA as discussed in [11]. During the segmentation task, every preprocessed test image is tested using the LS, CV, RG, and AC approaches. This test result confirms that the LS approach offered false result most of the time due to the visibility of CC. In most of the image cases, the CC pixel intensity is similar to the normal brain tissue intensity. Hence, for all the considered images, the extraction and evaluation task is implemented only with CV, RG, and AC.

**Figure 4:**
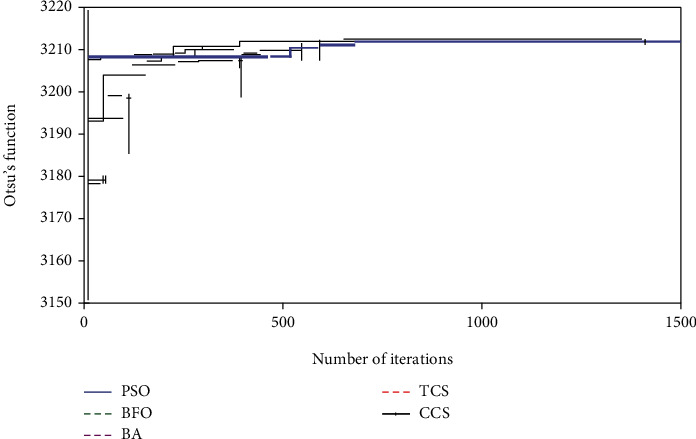
Convergence of the optimization search with Otsu's function.

Figure 4 represents the search merging of the heuristic algorithm for Otsu's trilevel threshold operation implemented on image 1. The proposed CCS is converged at 582^th^ iteration, and the search process is terminated at 1417^th^ iteration. This confirms that the projected CCS performs better compared to other approaches adopted in this study. Similar techniques are repeated with other threshold techniques, such as Kapur, Tsallis, and Shannon and for most of the cases, the proposed CCS offered improved outcome compared to the PSO, BFO, and BA. This confirms that the CCS works well for the chosen brain MRI thresholding problem.

**Figure 5:**
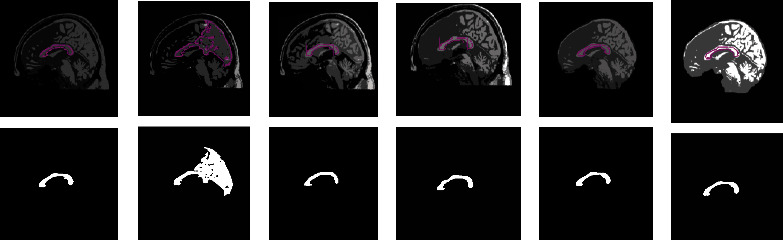
Extraction of CC using active contour segmentation: (a) Otsu's, (b) Kapur's, (c) Shannon's, (d) Tsallis, (e) Shannon's without the skull, and (f) Tsallis without the skull.

Figure 5 depicts the execution of the AC-based extraction of CC from the preprocessed test images presented in Figure 3. Similar procedure is recurrent for the additional 2D sagittal images of the database, and its effects are recorded. To confirm the preeminence of the considered preprocessing approach, a relative study among the mined CC and the GT is performed, and the image match and statistical outcomes are calculated. This comparative study confirmed that, for the chosen dataset, Otsu's and KE procedures are failed to provide better result compared to the SE and TE-based procedures. Hence, the results of Otsu's and KE are ignored, and the results of SE and TE are projected in this paper.  Table 1 represents the segmentation results attained for the representative images with the SE + AC and TE + AC. Similar results are attained with SE + CV, SE + RG, TE + CV, and TE + RG. Tables 2 and 3 present the similar information of the considered images and the statistical measures achieved during this experimental investigation. From Tables 2 and 3, it can also be observed that the outcome obtained with Otsu+AC and KE + AC is poor in comparison to the alternatives.

**Figure 6:**
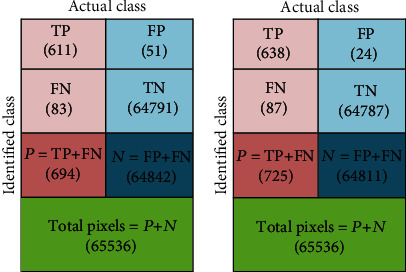
Confusion matrix to discuss the performance measure.

The performance of the projected CBDM is confirmed with a pixel level relative evaluation among the mined CC section and the GT. To demonstrate the performance, the mined CC sections SE + AC and TE + AC of image 1 are considered, and the obtained results are illustrated in Figure 6.  Figure 6(a) depicts the confusion matrix of SE + AC, and Figure 6(b) presents the confusion matrix of TE + AC. From these images, it can be distinguished that the image similarity constraints (ISP) offered by the proposed CBDM are better. Similar technique is repeated with further images, and the sample consequences obtained with image 1 to image 6 are depicted in Tables 2 and 3.

**Figure 7:**
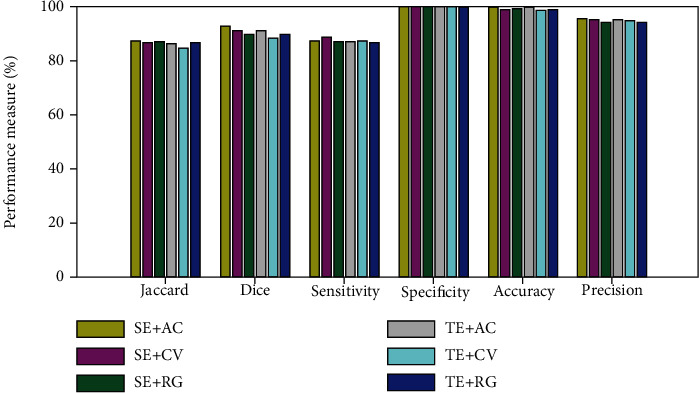
Performance evaluation of CBDM with chosen processing methods.

Table 3 authenticates that the image measures obtained with the SE are better when compared to TE. The average result computed for the ABIDE database (60 volunteers) in percentage is presented in Table 4, and its graphical representation is presented in Figure 7. This tabulation and figure confirm that the overall image similarity and the statistical outcomes obtained with SE + AC are superior compared with other approaches. This also authenticates that AC outperforms the CV and RG for the considered dataset.  Table 5 presents the computed values of TBA and CCA, and this result also authenticates that the average results of FCM + LS, SE + AC, and TE + AC are roughly identical.

**Figure 8:**
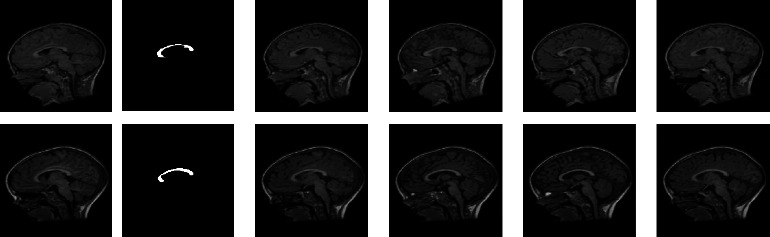
Sample test pictures and a sample result obtained with the MIDAS database.

**Figure 9:**
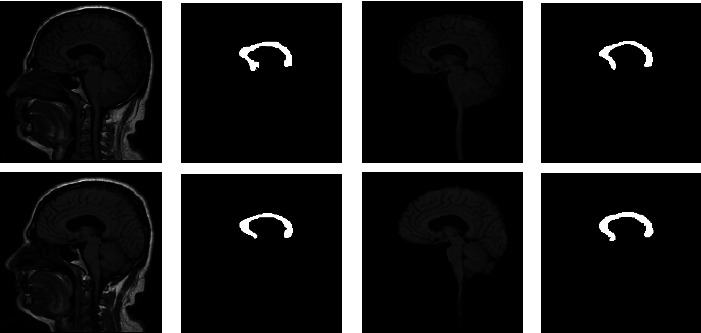
Sample 2D slices of real scan pictures and its corresponding result: (a) the sample test image with skull section, (b) segmented CC, (c) sample image without skull section, and (d) extracted CC from image (c).

The results depicted in Figures 8 and 9 also confirm that the method based on SE + AC and TE + AC provides better result on the MIDAS and Proscans datasets. From these outcomes, it can be understood that proposed CBDM has more efficacy in mining the CC segment from the T1 modality brain MRI slices.

This work also confirms that the average simulation period taken by SE + AC/TE + AC for ABIDE dataset is smaller (171.19 sec/168.94 sec) compared to other approaches (SE + CV = 192.16 sec, SE + RG = 174.28, TE + CV = 191.38, and TE + RG = 172.57 sec). The main limitation of the proposed technique is it implemented the semiautomated segmentation techniques, such as AC, CV, and LS procedures to mine the CC section. In future, the segmentation methods such as super pixel [66] and local binary pattern [67, 68] can be considered to extract the CC. Further, the planned method can be considered to evaluate the medical level brain MRI collected from volunteers who are associated with autism.

## 5. Conclusion

This paper suggested a computerized CC extraction tool with a two-step image processing scheme. The instigated method considers the blend of CCS-assisted trilevel thresholding with Shannon's/Tsallis entropy and segmentation based on the CV/RG/AC procedure. During the investigational assessment, the benchmark datasets, such as ABIDE and MIDAS, are used for the preliminary evaluation. Further, this tool is tested on the clinical 2D sagittal MRI of T1 modality obtained from a scan centre. The experimental investigation authorizes that proposed tool extracts the CC region from the brain picture with better accuracy and helps to compute the TBA and CCA for the 2D brain MRI. A comparative study also confirms that the results are approximately similar to the result existing in the literature with fuzzy *C*-means+LS procedure and better than multiphase LS. The proposed CBDM also offers better segmentation result for the clinical images. Hence, for the forthcoming requirements, this method can be considered in medical clinics to estimate the sagittal view MRI recorded with T1 modality. The proposed outcome of this work could be extended further to aid the investigations in identifying the prognosis of the disease at various stages.

## Figures and Tables

**Table 1:** Results obtained for the sample images.

Image (ASD)	Image	Test picture	GT	SE + AC	TE + AC
Male	Image 2	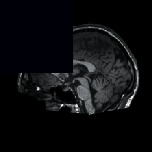	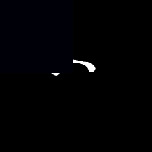	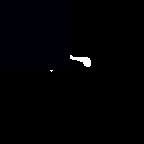	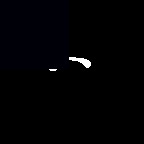
	Image 3	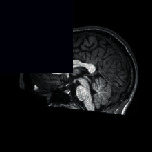	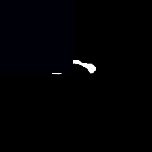	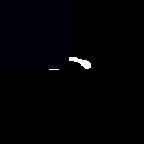	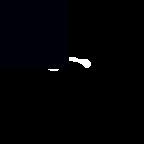
Female	Image 4	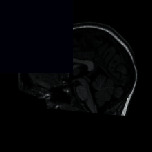	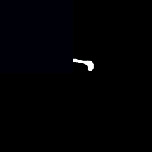	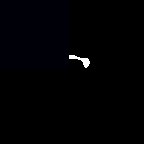	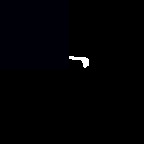
	Image 5	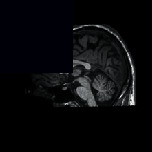	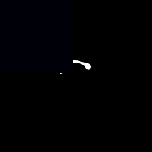	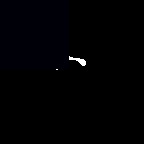	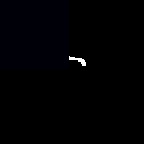
	Image 6	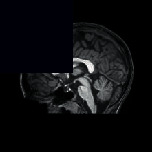	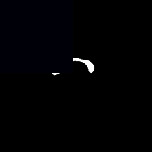	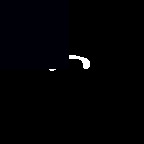	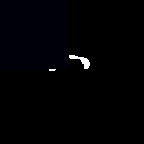

**Table 2:** Image similarity measures achieved for the selected sample mages.

Image	Method	TPR	FNR	TNR	FPR	Jaccard	Dice
Image 1	Otsu	0.8035	0.1965	1.0000	0.0000	0.8032	0.8909
	Kapur	0.1348	0.8652	0.9999	0.0001	0.1345	0.2372
	Shannon	0.8804	0.1196	0.9992	0.0008	0.8201	0.9012
	Tsallis	0.8800	0.1200	0.9996	0.0004	0.8518	0.9200
Image 2	Shannon	0.7907	0.2093	0.9992	0.0008	0.7454	0.8541
	Tsallis	0.8339	0.1661	0.9996	0.0004	0.8078	0.8937
Image 3	Shannon	0.8405	0.1595	0.9993	0.0007	0.7841	0.8790
	Tsallis	0.4450	0.1555	0.9993	0.0007	0.7876	0.8812
Image 4	Shannon	0.8912	0.1088	0.9991	0.0009	0.8281	0.9060
	Tsallis	0.8482	0.1581	0.9988	0.0012	0.7750	0.8732
Image 5	Shannon	0.8535	0.1465	0.9998	0.0002	0.8270	0.9053
	Tsallis	0.8261	0.1739	0.9995	0.0005	0.7705	0.8704
Image 6	Shannon	0.9133	0.9986	0.0014	0.0867	0.8019	0.8901
	Tsallis	0.8396	0.1604	0.9998	0.0002	0.8263	0.9049

**Table 3:** Image statistical outcomes attained for the selected sample images.

Image	Method	Sensitivity	Specificity	Accuracy	Precision
Image 1	Otsu	0.8035	1.0000	0.9976	0.9996
	Kapur	0.1348	0.9999	0.9373	0.9888
	Shannon	0.8804	0.9992	0.9980	0.9230
	Tsallis	0.8800	0.9996	0.9983	0.9637
Image 2	Shannon	0.7907	0.9992	0.9966	0.9287
	Tsallis	0.8339	0.9996	0.9976	0.9627
Image 3	Shannon	0.8405	0.9993	0.9978	0.9212
	Tsallis	0.8445	0.9993	0.9978	0.9212
Image 4	Shannon	0.8912	0.9991	0.9977	0.9212
	Tsallis	0.8482	0.9988	0.9969	0.8998
Image 5	Shannon	0.8535	0.9998	0.9987	0.9638
	Tsallis	0.8261	0.9995	0.9983	0.9197
Image 6	Shannon	0.9133	0.9986	0.9978	0.8680
	Tsallis	0.8396	0.9998	0.9979	0.9811

**Table 4:** Average values of similarity and statistical values of ABIDE dataset (60 volunteers).

Method	Jaccard	Dice	Sensitivity	Specificity	Accuracy	Precision
SE + AC	87.15	92.75	87.17	99.92	99.74	95.38
SE + CV	86.48	90.92	88.53	99.67	98.91	95.11
SE + RG	86.94	89.74	86.90	99.82	99.06	93.96
TE + AC	86.05	90.81	86.89	99.90	99.77	95.14
TE + CV	84.42	88.39	87.04	99.73	98.58	94.86
TE + RG	86.53	89.55	86.54	99.85	98.83	94.05

**Table 5:** Average values of TBA and CCA of ABIDE dataset (60 volunteers).

Parameter	FCM + LS [11]		Multiphase LS [9]		SE + AC		TE + AC	
	Controlled	ASD	Controlled	ASD	Controlled	ASD	Controlled	ASD
TBA	0.87	0.92	0.59	0.79	0.8104	0.8826	0.8685	0.9175
CCA	0.90	0.75	0.82	0.69	0.9092	0.7761	0.8917	0.7481

## Data Availability

The authors of this paper thank the contributors of the ABIDE and MIDAS database. The major part of the data considered to support the findings of this study is collected from these databases.
